# The Built Environment and Cognitive Disorders: Results From the Cognitive Function and Ageing Study II

**DOI:** 10.1016/j.amepre.2016.11.020

**Published:** 2017-07

**Authors:** Yu-Tzu Wu, A. Matthew Prina, Andy Jones, Fiona E. Matthews, Carol Brayne, on behalf of the Medical Research Council Cognitive Function and Ageing Study Collaboration

**Affiliations:** 1 Department of Public Health and Primary Care, Institute of Public Health, Forvie Site, University of Cambridge, School of Clinical Medicine, Cambridge, United Kingdom; 2 King’s College London, Institute of Psychiatry, Psychology and Neuroscience, Centre for Global Mental Health, Health Service and Population Research Department, London, United Kingdom; 3 Norwich Medical School, University of East Anglia, Norwich, Norfolk, United Kingdom; 4 Medical Research Council Biostatistics Unit, Institute of Public Health, University of Cambridge, Cambridge, United Kingdom; 5 Institute of Health and Society, Newcastle University, Newcastle upon Tyne, United Kingdom

## Abstract

**Introduction:**

Built environment features have been related to behavior modification and might stimulate cognitive activity with a potential impact on cognitive health in later life. This study investigated cross-sectional associations between features of land use and cognitive impairment and dementia, and also explored urban and rural differences in these associations.

**Methods:**

Postcodes of the 7,505 community-based participants (aged ≥65 years) in the Cognitive Function and Ageing Study II (collected in 2008–2011) were linked to environmental data from government statistics. Multilevel logistic regression investigated associations between cognitive impairment (defined as Mini-Mental State Examination score ≤25) and dementia (Geriatric Mental Status and Automatic Geriatric Examination for Computer-Assisted Taxonomy organicity level ≥3) and land use features, including natural environment availability and land use mix, fitting interaction terms with three rural/urban categories. Data were analyzed in 2015.

**Results:**

Associations between features of land use and cognitive impairment were not linear. After adjusting for individual-level factors and area deprivation, living in areas with high land use mix was associated with a nearly 30% decreased odds of cognitive impairment (OR=0.72, 95% CI=0.58, 0.89). This was similar, yet non-significant, for dementia (OR=0.70, 95% CI=0.46, 1.06). In conurbations, living in areas with high natural environment availability was associated with 30% reduced odds of cognitive impairment (OR=0.70, 95% CI=0.50, 0.97).

**Conclusions:**

Non-linear associations between features of land use and cognitive impairment were confirmed in this new cohort of older people in England. Both lack of and overload of environmental stimulation may be detrimental to cognition in later life.

## Introduction

Dementia and cognitive impairment in older age have been recognized as an important public health issue.^1^ Although a wide range of risk factors have been identified,^2^ prevention or risk-reduction strategies have focused largely on individual-level factors, such as lifestyle, health, and medical conditions.^3^ Potential environmental determinants have rarely been explored in existing studies or been taken into account during policy planning on dementia prevention or risk reduction.^4^, ^5^ As important environmental influences on lifestyle and health conditions have been widely recognized in public health research and used to develop potential interventions to promote individual and community health, aspects of the environment may also play a preventive role for cognitive disorders. In particular, recent studies have reported a higher prevalence of dementia in rural than urban areas,^6^, ^7^ together with an inverse relationship between cognitive function and area deprivation,^8^, ^9^, ^10^ which is typically taken to measure economic and material disadvantages (e.g., unemployment, low education, and household overcrowding)^11^ and widely used as a proxy of environmental conditions of local areas. This may suggest environmental characteristics at the small area level, usually defined as the community or neighborhood level, could have some influence on cognitive health.

Several built environmental features, such as land use mix, natural environment availability, and street connectivity, have been related to physical activity,^12^ depression,^13^, ^14^ and levels of social interaction,^15^ which are known risk or protective factors for dementia and cognitive decline.^2^, ^16^ The built environment may influence these lifestyle factors and increase cognitive reserve and general health throughout the life course. In addition to these potential indirect pathways, a recent review^17^ has suggested a direct association between environmental characteristics, sensory stimulation, and cognitive performance. Exposure to natural environment has been related to attention restoration,^18^, ^19^ whereas more-interactive environments, such as those with mixed land use, may provide a “brain training” setting and perceptual stimulation.^7^, ^17^ Counter to this is the potential overload of multiple stimulation caused by environmental stress in urban areas, which could have a negative effect on cognitive performance.^17^

The complexity of built environmental features in relation to cognition in later life has been reported in recent epidemiologic studies,^20^, ^21^ as well as in an earlier analysis using a follow-up investigation of the Medical Research Council Cognitive Function and Ageing Study (MRC CFAS).^22^ Based on 2,424 people aged ≥74 years across England in 2001, this earlier report suggests potential non-linear associations between cognitive impairment and features of land use, including natural environment availability and land use mix.^22^ Increased odds of cognitive impairment were found in both high and low levels of natural environment availability and land use mix. This might imply that both lack and overload of environmental stimulation could be detrimental to cognitive function in later life.

Environmental features at the small area level can vary greatly between urban and rural areas, with different meanings to residents.^23^ For example, most green space in rural areas is likely to be agricultural fields, which might not be suitable for recreation and physical activity. The heterogeneity of rural and urban contexts can influence interactions of older people with their local environments^7^ and thus the relationships between small area−level factors and cognitive function might be different in urban and rural settings.

The earlier MRC CFAS analysis was based on 10-year follow-up in 2001, focusing on survivors and responders from the baseline sample.^22^ The key findings from this work are described in Table 1, along with resultant hypotheses to be tested here. The earlier findings might have limitations relating to selection bias and could be outdated, given recent changes in dementia occurrence.^24^ The aim of this study is to examine whether findings from MRC CFAS can be replicated in the Cognitive Function and Ageing Study II (CFAS II), a new cohort starting from 2008 and representing the current older population in England. This paper further explores the potential for rural and urban differences in associations.

**Table 1:** Measurements of the Built Environment and Hypotheses to be Tested

**Environmental factors**	**Definition**	**Data sources**	**MRC CFAS findings**^a^	**CFAS II hypotheses**^b^
Land use mix	The diversity of land uses (domestic, green space, and commercial) in a defined area	Generalized Land Use 2001/2005	• A potential non-linear association between cognitive impairment and dementia and land use mix: the odds decreased from the first to the third quartile but then slightly increased in the fourth quartile.• A decreased odds of dementia in higher levels of land use mix after further adjusting for area deprivation.	• Land use mix has a non-linear association with cognitive impairment and dementia.• Outside conurbations, a higher level of land use mix is associated with lower odds of cognitive impairment and dementia.
Natural environment availability	Areas with natural vegetation, such as grass, trees, and plants	Generalized Land Use 2001/2005	• A potential non-linear association between cognitive impairment and dementia and natural environment: the odds decreased from the first to the third quartile but then slightly increased in the fourth quartile.	• There is a non-linear U-shaped association between natural environment availability and cognitive impairment and dementia.• In conurbations, higher availability of the natural environment is linearly associated with lower odds of cognitive impairment and dementia.

a Based on 2,424 people aged ≥74 years in England (survivors and responders to the 10-year follow-up in 2001).

b The current study based on 7,505 people aged ≥65 years in England (a representative sample of older people in England; baseline interview in 2008–2011). CFAS II, Cognitive Function and Ageing Study II; MRC CFAS, Medical Research Council Cognitive Function and Ageing Study.

## Methods

### Study Population

CFAS II is a population-based epidemiologic study of people aged ≥65 years in England. The primary purpose of the study is to investigate the epidemiology of dementia in the current UK older population and to explore changes in dementia prevalence and incidence over 2 decades. To compare the estimates with those from 1991 (MRC CFAS), CFAS II includes three of the original study centers in England (Newcastle upon Tyne, Nottingham, and Cambridgeshire) and used identical study designs and methods, apart from merged screen and assessment stages. The sampling frame is based on primary care registration including >2,500 community-based and institutionalized people with equal numbers of those aged 65–74 years and ≥75 years from each center. The baseline interviews (2008–2011) were delivered by trained interviewers using the standardized computerized interview in the participants’ residence. Full details of the study design and methods are published elsewhere.^24^

The total sample size of the CFAS II baseline was 7,796.^24^ The analysis here excluded 105 people who did not complete the interview but for whom a dementia diagnosis was derived from medical records and other relevant information. Because those living in care home settings might interact differently with their local environments, 185 people living in institutions were also excluded, together with one person aged 64 years. This left 7,505 for this study, comprising all the community-based participants across the three English centers. CFAS II was approved by relevant local research ethics committees and obtained informed consent from participants.^24^ This secondary data analysis does not require new IRB approval.

### Measures

Information on age, gender, and education was recorded at the interview. Education was divided into three groups: ≤9 years of education, 10–11 years, and ≥12 years.^25^ As several chronic conditions are related to cognitive disorders in older age,^2^ numbers of chronic illnesses, including vascular risk factors (hypertension, diabetes, stroke, heart attack, angina, and low blood pressure) and sensory impairment (hearing and vision impairment) were recorded based on self-reported information in the interview.

A structured assessment was used to measure cognitive function and mental status. Cognitive impairment was defined as a Mini-Mental State Examination score of ≤25, aligned with the previous CFAS II analysis.^25^ Dementia cases were defined as organicity level ≥3 using the Geriatric Mental Status and the algorithm of the Automatic Geriatric Examination for Computer-Assisted Taxonomy.^26^

Using the National Statistics Postcode Directory,^27^ postcodes of the CFAS II participants were mapped to Lower-layer Super Output Areas (LSOAs), a small geographic unit developed for the UK Census with an average of 1,500 residents per unit. For each LSOA, information for the Index of Multiple Deprivation 2010 and Generalized Land Use 2005 was obtained from the Neighborhood Statistics repository (www.neighbourhood.statistics.gov.uk) and linked to the CFAS II study areas.

Area deprivation was measured using the Index of Multiple Deprivation 2010, which summarized seven domains of characteristics related to deprivation (income, employment, education and training, health and disability, barriers to housing and services, living environment, and crime) based on data collected in 2007–2008.^11^ The Generalized Land Use 2005 data set provided areas of different types of land use in LSOAs and was used to calculate measures of land use mix and natural environment availability for the residential LSOA of each participant. The measure of land use mix was calculated based on literature,^28^ with a range from 0 (lowest heterogeneity of land use) to 1 (highest). A high level of land use mix indicates a close integration of different land uses, such as residential, commercial, and recreational areas. The measure of the natural environment availability was based on the percentage of green space and private gardens in each LSOA. The environmental measurements were divided into quintiles, aligned with the MRC CFAS analysis and UK Census reports.^9^, ^29^

The 2011 Rural/Urban Classification for Small Areas Geographies provided rural/urban categories for all the LSOAs in England.^30^ This analysis used three urban categories: Major Conurbation (mean population density [PD]=35.5 people per hectare), Minor Conurbation (PD=22.6), and City and Town (PD=16.5); and two rural categories: Town and Fringe (PD=5.9) and Village and Dispersed (PD=0.5).^31^ To increase the statistical power of the analyses, these categories were combined into three types: Conurbation (Major and Minor Conurbation), Urban City and Town, and Rural areas (Town and Fringe, Village, and Dispersed) based on the similarity of their environmental features.

### Statistical Analysis

Multilevel logistic regression was used to investigate the association between two environmental factors (land use mix and natural environment availability), and the outcomes of cognitive impairment and dementia before adjustment (Model 1) and then adjusted for individual-level factors (age, gender, education, and numbers of chronic illnesses) (Model 2). Further adjustment for area deprivation was conducted to control for the potential influence of socioeconomic disadvantage and other unmeasured related factors (Model 3). Given potential non-linear relationships, a likelihood ratio test was used to test for heterogeneity.

To investigate how associations might differ in urban and rural contexts, interaction terms between the two environmental factors and the rural/urban categories were included in regression models adjusting for individual-level factors. To retain adequate statistical power, the analysis focused on cognitive impairment only and the two environmental measures were re-categorized into tertiles, with the lowest tertile in Conurbation being the reference group. Data were analyzed in 2015 using Stata, version 12.0.

## Results

Distributions of individual-level factors are reported in Table 2. Among the 7,505 participants, the median age was 74 years (interquartile range, 11 years) and 54% were women. The prevalence of cognitive impairment and dementia increased with older age and lower education levels. Higher prevalence of cognitive impairment was found in women, those with two or more chronic conditions, and those living in rural areas, but these differences were not observed for dementia.

**Table 2:** Number and Percentage of Cognitive Impairment and Dementia Cases by Individual-Level Factors and Rural/Urban Categories

**Variable**	**Cognitive impairment**^a^	**Dementia**	**Total, n**
Participants	1,756 (23.7)	328 (4.4)	7,505
Missing	102 (1.4)	1 (0.0)	
Age group			
65–69 years	237 (12.4)	15 (0.8)	1,923
70–74 years	327 (17.7)	44 (2.4)	1,861
75–79 years	390 (24.9)	69 (4.3)	1,594
80–84 years	406 (33.3)	86 (7.0)	1,237
85+ years	396 (46.4)	114 (12.8)	890
*p*-value	**<0.01**	**<0.01**	
Gender			
Men	695 (20.3)	146 (4.2)	3,462
Women	1,061 (26.6)	182 (4.5)	4,043
*p*-value	**<0.01**	0.55	
Education			
≥12 years	189 (11.6)	36 (2.2)	1,644
10–11 years	779 (20.3)	129 (3.3)	3,871
≤9 years	765 (40.4)	147 (7.6)	1,946
*p*-value	**<0.01**	**<0.01**	
Number of chronic illness			
None	376 (21.4)	147 (8.0)	1,843
One	531 (22.6)	72 (3.1)	2,357
Two or more	849 (25.8)	109 (3.3)	3,305
*p*-value	**<0.01**	**<0.01**	
Rural/urban status			
Conurbation	1,088 (22.5)	207 (4.2)	4,905
Urban city and town	261 (25.3)	51 (4.9)	1,046
Rural area	407 (26.6)	70 (4.5)	1,554
*p*-value	**<0.01**	0.62	

*Note:* Values are *n* (%) unless otherwise noted. Boldface indicates statistical significance (*p*<0.05).

a Mini-Mental State Examination ≤25.

In Table 3, the associations between features of land use and cognitive impairment were not linear (Model 1) and these patterns persisted after adjusting for individual-level factors (Model 2). The odds decreased from the first to third quintile, but increased with higher levels of land use mix and natural environment availability. The lowest odds of cognitive impairment were found in the third quintile of land use mix (OR=0.69, 95% CI=0.56, 0.86) and natural environment availability (OR=0.81, 95% CI=0.67, 0.99). Although the associations with dementia did not achieve statistical significance, lower odds also appeared in the third or fourth quintile of land use mix and natural environment availability. After further adjusting for area deprivation, the odds of cognitive impairment and dementia were reduced in areas with high land use mix (Model 3). Living in areas with high land use mix was associated with 30% decreased odds of cognitive impairment (OR=0.72, 95% CI=0.58, 0.89). A similar reduction was observed for dementia (OR=0.70, 95% CI=0.46, 1.06), although this was not statistically significant.

**Table 3:** Unadjusted and Adjusted ORs of Cognitive Impairment and Dementia by Quintiles of Environmental Factors

**Environmental factors**	**Cognitive impairment**^a^			**Dementia**		
	**Model 1**^b^	**Model 2**^c^	**Model 3**^d^	**Model 1**^b^	**Model 2**^c^	**Model 3**^d^
Land use mix						
Q1 (lowest)	1.00	1.00	1.00	1.00	1.00	1.00
Q2	0.94 (0.74, 1.19)	0.86 (0.70, 1.06)	0.83 (0.69, 1.02)	1.02 (0.71, 1.45)	0.97 (0.67, 1.39)	0.90 (0.62, 1.30)
Q3	0.76 (0.60, 0.96)	0.69 (0.56, 0.86)	0.61 (0.50, 0.75)	0.97 (0.68, 1.39)	0.94 (0.65, 1.37)	0.83 (0.57, 1.22)
Q4	0.92 (0.73, 1.15)	0.76 (0.62, 0.93)	0.64 (0.52, 0.79)	0.87 (0.60, 1.25)	0.87 (0.60, 1.27)	0.70 (0.46, 1.04)
Q5 (highest)	1.06 (0.85, 1.32)	0.91 (0.75, 1.12)	0.72 (0.58, 0.89)	0.97 (0.68, 1.39)	0.93 (0.64, 1.34)	0.70 (0.46, 1.06)
*p*-value^e^	**0.03**	**<0.01**	**<0.01**	0.92	0.96	0.38
Natural environment						
Q1 (Lowest)	1.00	1.00	1.00	1.00	1.00	1.00
Q2	0.93 (0.76, 1.13)	0.89 (0.73, 1.07)	0.91 (0.75, 1.09)	0.89 (0.63, 1.25)	0.85 (0.59, 1.22)	0.88 (0.61, 1.27)
Q3	0.80 (0.65, 0.97)	0.81 (0.67, 0.99)	0.89 (0.74, 1.08)	0.72 (0.50, 1.03)	0.73 (0.50, 1.07)	0.81 (0.55, 1.19)
Q4	0.85 (0.69, 1.05)	0.94 (0.77, 1.14)	1.10 (0.90, 1.35)	0.95 (0.68, 1.33)	0.92 (0.65, 1.31)	1.07 (0.73, 1.58)
Q5 (Highest)	1.02 (0.81, 1.28)	1.17 (0.95, 1.44)	1.49 (1.20, 1.84)	0.86 (0.61, 1.22)	0.90 (0.62, 1.29)	1.12 (0.75, 1.68)
*p*-value^e^	0.13	**0.01**	**<0.01**	0.46	0.59	0.48

*Note*: Values are OR (95% CI) unless otherwise noted. Boldface indicates statistical significance (*p*<0.05).

a Mini-Mental State Examination ≤25.

b Model 1: Unadjusted model.

c Model 2: Adjusted for age, gender, education, and numbers of chronic illness.

d Model 3: Adjusted for age, gender, education, numbers of chronic illness, and area deprivation.

e *p-*value of test for heterogeneity. Q, quintile.

**Figure 1:**
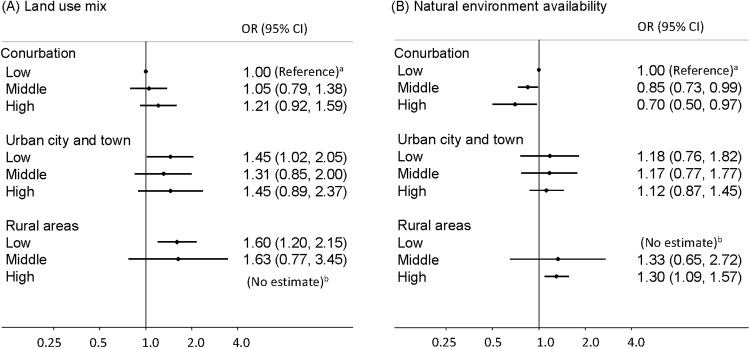
OR of cognitive impairment by interaction terms between land use mix, natural environment availability and rural/urban categories (estimates adjusted for age, gender, education, and chronic conditions). ^a^Reference group. ^b^Estimates were not available due to small sample sizes.

Figure 1 shows the associations between cognitive impairment and features of land use across the rural/urban categories. Two groups in rural areas did not have estimates owing to small sample sizes. Although the odds of cognitive impairment were slightly higher in rural areas than the reference group (the lowest tertile in Conurbation), the associations between cognitive impairment and land use mix were not substantially different across rural/urban settings. Living in areas with high natural environment availability was associated with up to 30% lower odds (OR=0.70, 95% CI=0.50, 0.97) of cognitive impairment in conurbations, whereas the associations were unclear in urban city and town areas and rural areas.

## Discussion

Building on the previous MRC CFAS analysis,^22^ this study used a new cohort of older people in England to investigate the associations between features of land use and cognitive impairment and dementia, and further explored potential urban and rural differences in more detail. This analysis further confirms the U-shaped associations that both high and low levels of land use mix and natural environment availability are associated with increased odds of cognitive disorders. After adjusting for individual-level factors and area deprivation, living in high land use mix areas was associated with a nearly 30% lower odds of cognitive impairment and dementia. The analysis of rural/urban differences shows a potential dose−response relationship between cognitive impairment and natural environment in conurbations. Despite overlapping 95% CIs for the middle and high tertiles, a 30% reduction in odds of cognitive impairment was observed for those living in areas of the highest natural environment availability.

The findings of non-linear relationships suggest that environments with especially low or high levels of land use diversity might be associated with a lack or overload of cognitive stimulation, and this could be detrimental to cognition in later life. Recent longitudinal studies in the U.S. have investigated features related to land use mix and also suggested their complex relationships with cognitive decline.^20^, ^21^ The Chicago Health and Aging Project including 6,518 people aged ≥65 years showed that living in a neighborhood with community centers and public transportation was associated with faster rate of cognitive decline over the 18-year observation period.^20^ This finding differs from an earlier study in Chicago that reported a positive association between cognitive function and neighborhood resources (libraries, recreational centers, and parks).^10^ Although a higher level of street integration and connectivity were both assumed to be representative of a more walkable environment, a small study (*n*=64) in Kansas reported differential associations between these two environmental factors and both baseline and change in cognitive function over 2 years.^21^ Although caution is needed in the interpretation of these factors in different sociopolitical and cultural contexts, these results might correspond to the present findings and suggest complicated interactions between the environment and cognitive stimulation.

Although mixed land uses could provide more-interactive environments for social and cognitively stimulating activities, areas with particularly high land use mix might also be associated with the presence of environmental stressors, such as noise, heavy traffic, and social disorder. These features could lead to overload of cognitive and sensory stimulation, overwhelming the potential benefits of being close to local services and resources.^17^ In this study, further controlling for area deprivation, a proxy of poor-quality environment, did attenuate the increased odds of cognitive impairment and dementia in the highest level of land use mix. Another possibility could be that features related to high land use mix might support individuals with cognitive impairment to continue living in their local communities. Alternatively, some older people could suffer from environmental stress in high land use mix areas but might not be able to move away because of economic disadvantage.^32^

The association between cognitive impairment and natural environment availability appears to differ in urban and rural settings. Though exposure to green space can be beneficial to psychological restoration,^18^, ^33^ rural areas with very high natural environment availability may have more social isolation^34^ and a consequent lack of cognitive stimulation. In contrast to rural areas, a linear relationship was found in conurbations. In addition to the beneficial influence on physical activity,^12^ green space in urban settings has been suggested to buffer against stress^33^ and might also reduce stimulation overload.^17^

### Limitations

This study was based on a multicenter population-based cohort of a current older population in England, including participants from a wide variety of sociodemographic backgrounds and environmental contexts. Cognitive assessment and dementia diagnosis were based on a structured interview to avoid potential variation in diagnostic standards. Further, the data set was generally complete with a low percentage of missing data (<2%).

Given the cross-sectional nature of the data, the ability to determine causality is limited and reverse causality is possible, as older people might need to change their residence to receive care from family members or health services as a result of poor cognitive and functional abilities. Unfortunately, information on relocation in recent years was not available in CFAS II. Nevertheless, 95% of the cohort reported that they had lived in their local area for more than 5 years. Although the same area may not equate to exactly the same address, this suggests relocation bias may be minimal. Some environmental factors such as traffic intensity could be potential confounding factors but they were not adjusted in the analysis, owing to lack of available data. Although a number of lifestyle and social engagement measures are available for the cohort, they are relatively simple. Given that this analysis is cross-sectional and that the potential role of factors such as lifestyle is unclear, this study did not investigate them further, as potential mediation and moderation are better investigated in future longitudinal research with appropriate follow-up measurements.

The LSOAs in rural areas (median, 11,500 m^2^) were much bigger than those in urban areas (median, 350 m^2^), but variations in environmental factors were generally small across geographic units. Skewed distributions of environmental factors in rural areas caused small sample size for some interaction terms and insufficient power to test urban/rural differences. Further, boundaries of LSOAs might not reflect the actual activity space of those living in a community. Although this study included >7,500 people, the low prevalence of dementia limits statistical power to detect variation across quintiles.

## Conclusions

The findings of this study reinforce the earlier observed association between environment and cognition in later life. Policy planning on dementia prevention or risk reduction may consider aspects of environment and address, such population-level determinants. In recent years, several policies around aging and well-being have started to focus on creating supportive environments for health.^35^ Although high land use mix and natural environment availability have been suggested to support active and healthy aging^12^, ^35^ instead of emphasizing a unidirectional impact of certain environmental features, achieving a balance between support and stimulation from local environments could be particularly important for cognitive health in older people. Features related to a walkable environment seem to have unexpected associations with cognition in older age. Population-based longitudinal studies are needed to clarify causal directions and investigate underlying mechanisms considering both direct and indirect pathways via physical activity, social interactions, and other potential mediators. Future studies may also consider the quality and types of green space,^36^ as these may provide insights into urban/rural differences in observed associations.
